# Communication in Cancer Care in Asia: A Narrative Review

**DOI:** 10.1200/GO.22.00266

**Published:** 2023-06-26

**Authors:** Masanori Mori, Cheng-Pei Lin, Shao-Yi Cheng, Sang-Yeon Suh, Sayaka Takenouchi, Raymond Ng, Helen Chan, Sun-Hyun Kim, Ping-Jen Chen, Kwok Keung Yuen, Maiko Fujimori, Takashi Yamaguchi, Jun Hamano, Yoshiyuki Kizawa, Tatsuya Morita, Diah Martina

**Affiliations:** ^1^ Division of Palliative and Supportive Care, Seirei Mikatahara General Hospital, Hamamatsu, Japan; ^2^ Institute of Community Health Care, College of Nursing, National Yang Ming Chiao Tung University, Taipei, Taiwan; ^3^ Cicely Saunders Institute of Palliative Care, Policy and Rehabilitation, Florence Nightingale Faculty of Nursing, Midwifery, and Palliative Care, King's College London, United Kingdom; ^4^ Department of Family Medicine, College of Medicine and Hospital, National Taiwan University, Taipei, Taiwan; ^5^ Department of Family Medicine, Dongguk University Ilsan Hospital, Goyang, South Korea; ^6^ Department of Medicine, Dongguk University Medical School, Seoul, South Korea; ^7^ Department of Nursing Ethics, Division of Human Health Sciences, Graduate School of Medicine, Kyoto University, Kyoto, Japan; ^8^ Palliative and Supportive Care, Woodlands Health, Singapore, Singapore; ^9^ The Nethersole School of Nursing, Faculty of Medicine, The Chinese University of Hong Kong, Hong Kong, China; ^10^ Department of Family Medicine, School of Medicine, Catholic Kwandong University, International St Mary's Hospital, Incheon, South Korea; ^11^ Department of Family Medicine, Division of Geriatrics and Gerontology, Kaohsiung Medical University Hospital and School of Medicine, Kaohsiung Medical University, Kaohsiung, Taiwan; ^12^ Marie Curie Palliative Care Research Department, Division of Psychiatry, University College London, London, United Kingdom; ^13^ Department of Clinical Oncology, Queen Mary Hospital, Hong Kong, China; ^14^ Division of Supportive Care, Survivorship and Translational Research, National Cancer Center Institute for Cancer Control, Tokyo, Japan; ^15^ Department of Palliative Medicine, Kobe University Graduate School of Medicine, Kobe, Japan; ^16^ Department of Palliative and Supportive Care, Faculty of Medicine, University of Tsukuba, Tsukuba, Japan; ^17^ Department of Medical Oncology, Erasmus MC Cancer Institute, University Medical Centre Rotterdam, Rotterdam, The Netherlands; ^18^ Department of Public Health, Erasmus MC, University Medical Centre Rotterdam, Rotterdam, The Netherlands; ^19^ Department of Internal Medicine, Division of Psychosomatic and Palliative Medicine, Faculty of Medicine Universitas Indonesia, Jakarta, Indonesia; ^20^ Cipto Mangunkusumo National General Hospital, Jakarta, Indonesia

## INTRODUCTION

Despite the advancement of modern medicine, cancer remains one of the leading causes of death across the globe.^1^ Patients with cancer may react to diagnostic, prognostic, and treatment information with negative emotions such as fear, denial, and anger.^2^ Thus, effective communication between health care providers (HCPs) and patients as well as their families is essential to build rapport, help patients cope with their illnesses, convey adequate information, address their concerns, and achieve individualized care through shared decision making.

**CONTEXT**

**Key Objective**
How do Asian cultures influence communication with patients with cancer and their families?
**Knowledge Generated**
This narrative review highlights that Asian patients tend to value harmony in family relations over individualistic autonomy and that communication in cancer care in Asia is characterized by a reluctance to tell the truth, implicit communication, and family-centered decision making styles. However, recent research has shown a gradual shift toward open communication in major themes that include cancer diagnosis, prognosis, advance care planning, and end-of-life discussions.
**Relevance**
Culturally sensitive, effective strategies for communication with patients with cancer and their families are of utmost importance in Asia. Future efforts are needed to obtain more insight into intra- and intergroup differences in Asia and other parts of the world.

Communication can be heavily influenced by culture.^3^ The American Society of Clinical Oncology Clinical Guidelines strongly recommends that HCPs should explore how a patient's culture affects their end-of-life (EOL) decision making or care preferences.^2^ Understanding cultural norms and unique practice patterns may help HCPs improve the quality of care through sensitive and individualized communication.^2^ Asia harbors more than half of the world's population and has much cultural diversity.^4^ As approximately half of global cancer cases occurred in Asia in 2020,^1^ it is important to understand the current status, controversies, and future directions of communication in cancer care in Asia. To the best of our knowledge, there has been no review highlighting various topics of communication with patients with cancer in east and southeast Asia. In this narrative review, we provide an overview of communication in cancer care in Asia, with a particular focus on countries and regions in the east and southeast Asia.

## CULTURAL CONSIDERATIONS IN ASIA

### Traditional Cultural Values Associated With Truth-Telling and Decision Making Styles

Over the past few decades, studies have revealed cultural differences in attitudes toward truth-telling and decision making styles.^3,5,6^ Traditionally, Asian people have general attitudes against truth-telling and preferences for a family-centered decision making style^3,5,6^ (Table 1). In high-context cultures, such as in Asian, mutual expectations and feelings within the social context are implicit and not explicitly expressed.^6^ Frank communication can often be considered impolite; people tend to say what they really want to convey more implicitly and expect others to assume their feelings and act accordingly.^6^ Moreover, Asian patients tend to value harmony in family relations over absolute autonomy and defer decision making to families and HCPs.

**TABLE 1:**
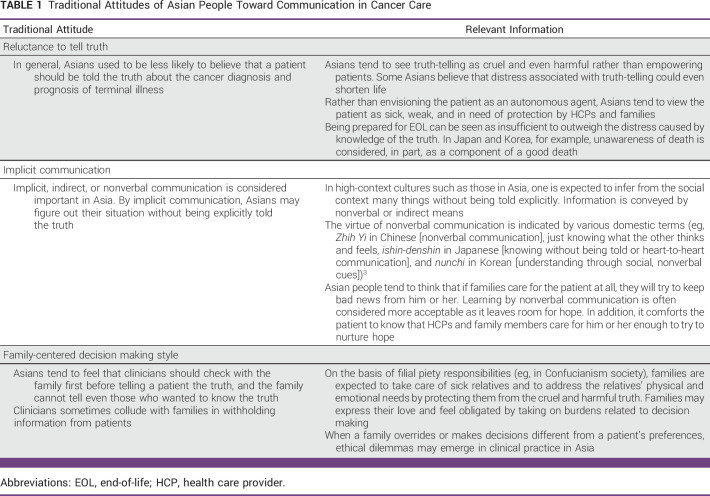
Traditional Attitudes of Asian People Toward Communication in Cancer Care

In recent years, however, Asian people's preferences have gradually shifted toward more open communication, in part due to the effects of globalization of liberal values.^7–9^ A significant proportion prefers truth-telling, explicit communication, and patient-centered decision making approaches.^10^ Thus, the assumption that Asian patients do not want open communication or the authoritarian and paternalistic behaviors of some physicians can hamper shared decision making as patients may feel that they are not respected or heard.^11^ It is important to note that this is a matter of relative emphasis, and assessment of the informational needs of individual patients and families is essential. HCPs should avoid the dual pitfalls of cultural stereotyping or ignoring the potential influence of culture and acculturation.^3,5,6^

### Religion and Communication

Under stressful circumstances (eg, when receiving a serious illness diagnosis), some people turn to religion as their coping mechanism.^12^ Illness, for instance, is seen as part of a divine plan. Religious practices (eg, prayer or meditation) enhance a sense of control over stressful events by helping individuals achieve a personal relationship with a higher entity that offers strength and support to cope with their illness.^12^

Seventy-nine percent of Asians are religious (25% Hindus, 24% Muslims, 11% Buddhists, and 7% Christians).^13^ Religious beliefs can affect individuals' engagement in cancer care communication by influencing their readiness to engage in future care planning and attitudes in decision making.^7,14,15^ With regard to information needs, individuals who believe that God predetermines life would not always appreciate information about estimated life expectancy.^16^ Religious beliefs such as beliefs in miracles have been shown to heavily influence prognostic understanding.^16^ Buddhists believe in the natural life process of birth, aging, illness, and death.^17^ Therefore, they believe in the predestination of the life circle and are reluctant to discuss with HCPs about EOL care issues, let alone make decisions regarding the extent of medical treatment. In addition, a sense of fatalism and preference to focus on here and now have limited one's ability to engage in future planning.^7^ Buddhists may prefer to defer decision making and treatment outcomes to their family members, the medical team, or even supreme gods.^18^ Thus, information provision without carefully considering patients' preferences may disrespect patients' values and religious beliefs. Accordingly, a thorough assessment of which information is preferred by and could be helpful for patients is an important step before medical information disclosure.

HCPs should be well-conversant of general principles, in particular, religions, when engaging in serious illness communication with patients with cancer. For instance, understanding the Islamic principle of *tawakkul*, or placing one's entire trust and reliance on God's plan, means that advance care planning (ACP) should be introduced as a process to create connection with individuals and their families and prepare them and their loved ones for future scenarios rather than merely formulating a plan ahead of time. Similarly, when discussing treatment options with Buddhists or Hindus, understanding of the life cycle, karma, and samsara—the belief that their actions in past lives predetermine their current physical suffering—is necessary.^19,20^ Patients with such beliefs might consider that undergoing physical suffering at EOL could reverse negative karma and mean that the departing soul will experience less suffering in the next life.^19^ In such instances, symptom relief should be offered while being open to accommodating a patient's wish to not pursue symptom management. Studies have shown that, besides involving religious leaders or interpreters, religious terms are also helpful in addressing medically obscure concepts, such as using the term *mudharat* (or *harm* in Islam) when discussing medical futility.^15^ The use of the specific Hindu terms *aatman* (or *soul* in Hindu) and *gangajal* (or holy water) helps facilitate connection with Hindu patients and their families during EOL discussions by showing acceptance of their religious beliefs and customs.^19^ Finally, we suggest that HCPs should develop cultural humility, which involves taking whatever efforts are needed to foster a meaningful understanding of a particular religion's common features while avoiding stereotypical characterization.

## MAJOR COMMUNICATION THEMES THROUGHOUT THE DISEASE TRAJECTORY

### Cancer Diagnosis and Treatment

Disclosing cancer diagnosis to patients and their family members is a distressing experience and can be challenging to physicians. Breaking bad news requires trusting relationships between patients, family, and physicians, skilled communication strategies such as appropriate timing and cultural sensitivity, and the ability to harness further support for the patient. Evidence has strongly supported that the acceptance of cancer diagnosis disclosure among patients highly depends on existing social norms, cultural values, local relevant legislations, and perception of autonomy.^5^

Diagnosis nondisclosure to patients has traditionally been widespread in clinical practice in Eastern cultures (family-oriented autonomy, such as in Japan,^21^ Taiwan,^22^ and Korea^6^) although evidence shows that informing patients with cancer of their diagnosis might not have a detrimental impact on their quality of life.^23^ Family caregivers commonly request the physicians to conceal the cancer diagnosis from patients, while physicians tend to inform the bad news to the next of kin before telling the patients. The belief is that this practice would protect the patients from physical and psychological distress, which might inadvertently hasten to death.^24^ Although still widely practiced, this dilemma in truth-telling also engenders substantial moral distress among HCPs.^25^ However, in some regions, there is a clear shift toward diagnostic disclosure in recent decades. In Japan, for example, the proportion of patients with cancer who were informed of their diagnosis increased from approximately 14% in the 1980s to 74% in 2012 and over 90% in 2016.^26^ Throughout this period, preferences of adult patients with cancer regarding the disclosure of bad news were clarified on the basis of which culturally adaptive communication skills training (CST) was developed.^27,28^ In addition, preferences specific to giving adolescent and young adult patients bad news related to cancer diagnosis and treatment have recently been explored in Japan.^29^ These included communicating in a way that considers their age and cognitive development, mentioning generation-specific social factors, not showing excessive empathy, and communicating in a way that supports their decision making.

In Asia, the use of complementary and alternative medicine (CAM) including spiritual healing practice is highly prevalent and it has various implications to decision making.^30–32^ While some evidence exists in the effects of CAM on various symptoms related to cancer and its treatment, patients who use CAM often refuse other conventional cancer treatment and can have a higher risk of death than those who do not use CAM.^33,34^ In particular, patients of low socioeconomic status may first approach the traditional healers with their medical problems, and only after failure of such treatment did they move to physicians for conventional therapies.^31^ Notably, it has been reported that more than half of patients with terminal illnesses conceal its use to HCPs.^30,35^ Thus, the common application of CAM in Asia can affect the relationship between patients and HCPs.^36^

Previous studies in Asia indicated that a need for information about CAM was frequent for various cancer types and the majority of oncologists would initiate a discussion on CAM use.^37,38^ However, patients with cancer and oncologists may hold discrepant views on CAM. For example, a survey in a general hospital showed that patients with cancer were more likely to believe that CAM was effective, whereas oncologists had more concerns about adverse events of CAM use, and that oncologists usually discouraged their patients from using CAM.^38^ Such discrepancies could hamper mutual trust without effective communication. It may be helpful for HCPs to be mindful of meaning of care practices in CAM, which include an additional beneficial choice for health as it fulfills patients' needs and it is viewed as the way of returning to nature and emotional psychological healing as the patient may be encouraged by surrounding people and feel calm and peaceful when using CAM.^35,39^ As Asians have diverse values and preferences for CAM, HCPs should establish an open communication model, encourage patients to share CAM experiences, and provide evidence-based information on the use of CAM practice to improve patient satisfaction and reduce the potential damage caused by harmful use.^30^

### Incurability and Prognosis

Sensitive discussions of incurability and prognoses with patients with advanced cancer are among the top priorities. Yet, such conversations remain challenging for HCPs.^2^ A multicenter, prospective cohort study in the United States revealed that 69% of patients with metastatic lung cancer and 81% of those with metastatic colorectal cancer did not report understanding that chemotherapy was not at all likely to cure their cancer.^40^ Another US cohort study involving 590 patients with metastatic cancer demonstrated that 71% wanted to be told their life expectancy, but only 17.6% recalled a prognostic disclosure by their physician.^41^ Among patients willing to estimate their life expectancy, those who recalled prognostic disclosure were offered more realistic estimates as compared with patients who did not, showing the difficulty and importance of prognostic communication.^41^ A Japanese survey indicated that only 39% and 18% of patients with advanced cancer recognized their incurability and prognosis, respectively.^42,43^ Traditionally, physicians tend to disclose the prognosis to families instead of patients in Asia^5,44^ or only discuss the prognosis when prognostic disclosure is requested.^45^ In a systematic review, Asian patients were shown to prefer that relatives be present when receiving bad news more than Westerners and desire discussing their life expectancy less than Westerners.^46^ However, recent studies in Asia have suggested a gradual increase in the proportion of patients who are aware of their prognosis and incurability and/or prefer communication on these topics with their physicians.^47^ A longitudinal study in Taiwan demonstrated that about 60% of terminally ill patients with cancer had accurate prognostic awareness.^48^ A randomized controlled trial involving patients with advanced cancer in Taiwan also showed that an individualized, interactive intervention promoted patients' prognostic awareness and reduced futile medical treatment.^49^ A cohort study in Korea revealed that around 80% of patients preferred to be informed of their terminal status.^50^ Recently, a randomized, video vignette study conducted in Asia indicated that explicit prognostic disclosure could lead to greater satisfaction in patients without triggering anxiety.^51^ Japanese patients with cancer preferred explicit prognostic information—the median survival, typical range, and best/worst cases—than nondisclosure or implicit communication in a cross-sectional survey.^52^ These studies show that although the gap between patients' desire for prognostic disclosure and communication practices of physicians is common to both Western and Asian cultures, Asian patients and physicians may be more reticent. However, attitudes seem to have shifted over the years in some Asian countries.

As patients' values and preferences for information vary, every person should be treated as an individual without a priori being attributed to the stereotypes of his or her own culture.^53^ HCPs should build rapport with patients and families to explore their readiness and information needs. Tailored communication of prognoses would enhance patients' quality of life in their limited time.

### ACP

ACP, as a process that enables individuals to define and discuss goals and preferences for future medical treatment and care with family and HCPs and to record and review these preferences if appropriate,^54^ is not widely practiced in Asia.^8^ Studies in Asia have demonstrated low awareness of and engagement in ACP among both people in the community and those diagnosed with advanced illness.^7,8,55^ In addition, systematic reviews evaluating age-appropriate ACP and related factors in children diagnosed with a life-limiting condition did not identify studies conducted in Asia, whereas cross-cultural adaptation of an ACP communication guide for Chinese adolescent and young adults has recently been reported in a domestic journal.^56–58^

Because of their strong trust in families and/or HCPs or their desire to avoid relational conflicts, Asian patients often prefer their family and/or HCPs to make decisions on their behalf.^7,59^ However, patients are often ill-informed about their illness, which hinders them from further reflections on the needs for ACP.^7,8,60^ Adoption of ACP has been demonstrated to vary between different countries and regions in Asia and cultures within a single country/region, highlighting the deep influence of culture on readiness for ACP.^61^

Paradoxically, although Asian HCPs recognize the importance of ACP, they rarely engage the patient in ACP, and late initiation of EOL conversations is the norm.^7,8^ Compared with Western HCPs, Asians tend to give greater voice to patients' families in ACP.^8^ Barriers to ACP include HCPs' lack of knowledge and skills in effective communication, fear of conflict with patients' families, emotional barriers toward having such challenging conversations, and the lack of a standard system for ACP.^7,8^

Key recommendations include education and engagement of both the public and HCPs to raise awareness, dispel misconceptions, build capacity, and develop institutional support for ACP.^7,8,62^ There are also calls to develop culturally attuned approaches in ACP that take into account an individual's readiness and religious beliefs, communication norms, and the role of the family and physician.^7,8,15,63–65^ Novel approaches to ACP in Asian groups such as the use of culturally tailored conversation cards,^66^ the development of a palliative care needs screening tool as a trigger for offering ACP,^67^ community-based models,^68^ and the implementation of culturally adapted intervention^69^ have shown promise. Where systematic training of HCPs, physician leadership, and institutional support were present, there was enhanced adoption of ACP.^70,71^

### Other EOL Discussions

Multiple studies showed that early discussions about EOL, or goal-of-care conversations, are associated with reduced use of aggressive yet futile treatment near death, provision of EOL care consistent with patients' preferences, and improved patients' quality of life.^72^ EOL discussions with patients with cancer include, but are not limited to, hospice, place of death, code status, and the possibility of impending death (ie, last weeks to days of life).^10,45^

A nationwide survey of medical oncologists in Japan indicated that they would discuss EOL issues later in the disease trajectory.^45^ Only 14%, 9.8%, and 4.2% of Japanese oncologists would discuss hospice, place of death, and Do-Not-Resuscitate (DNR) status, respectively, at diagnosis with a hypothetical patient with newly diagnosed metastatic cancer.^45^ The majority of physicians would defer such discussions to when there is no more anticancer treatment or only if the patient is hospitalized. Overall, physicians perceiving greater importance of life completion in experiencing a good death and less discomfort in talking about death were more likely to have EOL discussions at diagnosis.^45^ Discussions about ending anticancer treatment and transitioning to palliative care can also be difficult. However, most patients preferred physicians to be realistic about their likely future and listen to their distress and concerns and wanted to be reassured that their symptoms would be controlled.^73^ Patients with cancer in Asia also prefer reassuring statements when HCPs discuss EOL issues.^74^ These include the additional statement of hope for the best, and prepare for the worst when communicating prognosis and the assurance of symptom control when discussing DNR.^74^ When introducing the possibility of hospice referral, it is also beneficial to share a specific goal of the referral and to give assurance of continuity of care and nonabandonment.^74^

A recent East Asian study involving patients with advanced cancer who died in palliative care units revealed that 4.8%, 19.6%, and 66.4% of patients were explicitly informed of their impending death by their physicians in Japan, Korea, and Taiwan, respectively, whereas more than 90% of families were informed across all the regions studied.^10^ These findings not only indicate that explicit communication about impending death with patients is not necessarily the norm in Asia but also demonstrate that various practice patterns do exist in East Asia.

Finally, EOL discussions may contribute to positive family outcomes in Asia. A bereaved family survey showed that earlier EOL discussions between families and physicians were associated with a better family-perceived quality of death and EOL care and a lower frequency of depression and complicated grief during bereavement.^75^

## STRATEGIES TO IMPROVE COMMUNICATION

### CST

Given the challenges of communication in cancer care, a structured approach to facilitate the communication process has been widely advocated. Among the most widely used approaches to bad news telling is the mnemonic approach toward bad news telling: Setting, Perception, Invitation, Knowledge, Emotions, Strategy and Summary (SPIKES) protocol.^76^ Holmes and Illing proposed the mnemonic tool including six stages: acknowledge the request for nondisclosure, build the relationship, find common ground, honor the patient's preferences and outline the harm of nondisclosure, provide emotional support, and devise a supportive solution (ARCHES) tool with intention to use before SPIKES in a situation where the family has requested nondisclosure of the diagnosis to a patient.^25^ The key mechanism of action is to first acknowledge the request, then build up a relationship to explore the common ground of such a request followed by honoring the patient's right to know and outlining the potential harm of nondisclosure, and finally provide emotional support to the family and devise a future care plan. Implementation of such a framework may help realign patients and family caregivers' expectations on cancer diagnosis while respecting cultural norms.^25^

The CST embedded in Respecting Choices, an ACP program developed in the United States, was first introduced in Asian regions, including Singapore and Hong Kong, in the 2000s.^77,78^ Recently, VitalTalk and Serious Illness Care Program, two evidence-based CST initiatives also founded in the United States, have been adapted in Japan and Hong Kong.^79,80^ Multiple pedagogies were used for training, including didactic lectures, demonstration videos, role plays, and simulations.

Apart from adapting communication models from Western culture directly, culturally sensitive models also emerged over these years. For example, a SHARE model developed in Japan for facilitating compassionate bad news telling has been adopted in Taiwan, Mainland China, and Korea.^28,81,82^ Moreover, CST is no longer limited to physicians or nurses working in oncology settings, but has expanded across disciplines, clinical specialties, and care settings, for example, Education for Implementing End of life Discussion (E-FIELD) in Japan and online learning modules in the Jockey Club End-of-Life Community Care Project in Hong Kong.^67^ Evidence has generally shown that CST can effectively improve HCPs' knowledge and confidence in communicating the prognosis and planning for future care with their patients.^81,83,84^

### Other Tools Supporting Communication

Among the most effective and well-used tools to support patient-HCP communication are question prompt lists (QPLs). QPL can guide HCPs to use helpful questions and statements, while patients consistently perceive QPLs as helpful.^85^ The effectiveness of QPL has been validated in East Asia, including Japan,^86^ Singapore,^87^ and Taiwan.^88^

Moreover, various patient-reported outcomes (PROs) measures have been used to facilitate communication in Asia and internationally.^89^ Stakeholder engagement is recommended to strengthen the inclusion of PRO into routine practice to involve patients in shared decision making and care planning systematically.^89^ HCPs in Asia must also be vigilant in recognizing that PROs are only effective when information is given to the physician during consultations.^90^

While tools for decision support and goals-of-care discussions may be effective in improving the quality of patient-HCP communication in Asia, strategies such as the life-line interview method (ie, an integrative method for eliciting in-depth autobiographical information about life history and future expectations related to the emotions of each significant life event in an individual's life)^91^ to engage patients in exploring their values should be incorporated to overcome difficulties experienced by HCPs.^8^ Finally, strategies to overcome limitations in current practices when family members request nondisclosure of bad news to their loved one include the following: exploring reasons of family encouraging nondisclosure; flipping the roles of the patient and the family and asking what the family members would want if they were the patients and explaining what the patient may want to do with proper disclosure; discussing values, goals, and preferences by addressing patients' and family members' concerns and emotions; and facilitating communication between patients and family members.^92^ Holding a family conference involving both the patient and key persons is also beneficial.^93^ All these may promote mutual understanding and shared decision making between the patient and family members regarding future medical treatment and care, while respecting values, goals, and preferences of both parties.

## CONTROVERSIES AND FUTURE DIRECTIONS

Despite the increasing number of studies on communication in cancer care in Asia, limitations and controversies exist in the literature (Table 2). Evidently, there is no one-size-fits-all approach in communication, and the field is evolving markedly. Table 3 summarizes future directions in this field. Future studies should involve both the East and West and clarify intra- and intergroup differences in perceptions and practice regarding communication in cancer care. As the burden of cancer care rises in Asia, there is an urgent need to develop effective, culturally sensitive, and individualized communication strategies to enhance shared decision making and person-centered care.

**TABLE 2:**
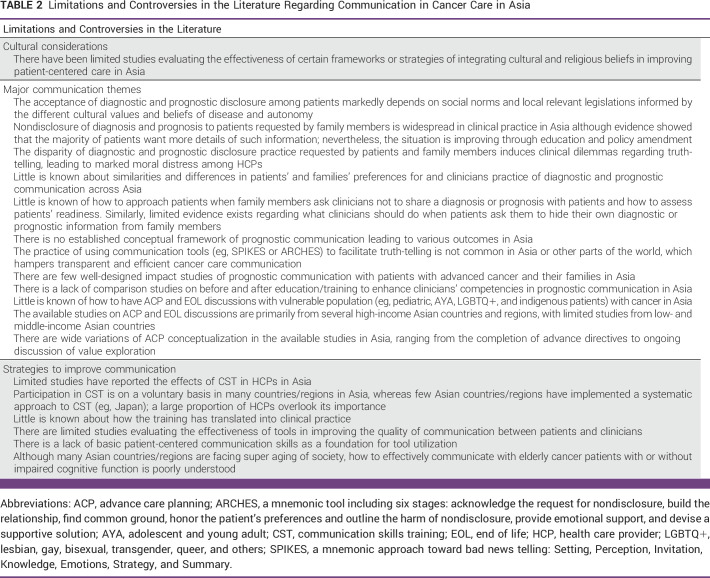
Limitations and Controversies in the Literature Regarding Communication in Cancer Care in Asia

**TABLE 3:**
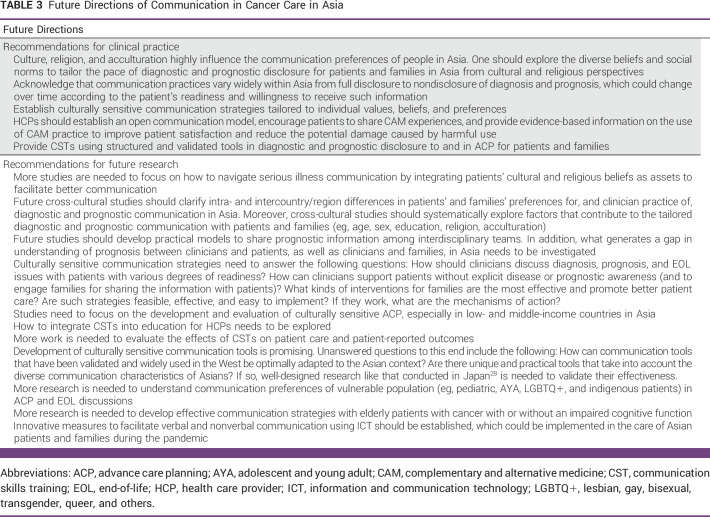
Future Directions of Communication in Cancer Care in Asia
